# Interaction between Fokl polymorphism and vitamin D deficiency in the symptoms of mental disorders in adults: a population-based study

**DOI:** 10.1038/s41598-024-57558-1

**Published:** 2024-03-22

**Authors:** Thaís da Silva Sabião, Luiz Antônio Alves de Menezes-Júnior, Aline Priscila Batista, Samara Silva de Moura, Adriana Lúcia Meireles, Mariana Carvalho de Menezes, George Luiz Lins Machado-Coelho, Júlia Cristina Cardoso Carraro

**Affiliations:** 1 School of Nutrition, Postgraduate Program in Health and Nutrition, Research and Study Group on Nutrition and Public Health (GPENSC), Federal University of Ouro Preto, Campus Morro do Cruzeiro, Ouro Preto, MG 35400-000 Brazil; 2 Postgraduate Program in Biological Sciences, Federal University of Ouro Preto, Campus Morro do Cruzeiro, Ouro Preto, MG 35400-000 Brazil; 3 Department of Clinical and Social Nutrition, Research and Study Group on Nutrition and Public Health (GPENSC), School of Nutrition, Federal University of Ouro Preto, Campus Morro do Cruzeiro, Ouro Preto, MG 35400-000 Brazil; 4 Epidemiology Laboratory, Medical School, Federal University of Ouro Preto, Campus Morro do Cruzeiro, Ouro Preto, MG 35400-000 Brazil

**Keywords:** Psychiatric disorders, Risk factors

## Abstract

Mental disorders are intricate and multifaceted and encompass social, economic, environmental, and biological factors. This study aimed to explore the potential association between vitamin D deficiency and anxiety and depression symptoms in adults, considering the role of the vitamin D receptor gene polymorphism FokI (rs2228570). This was a population-based cross-sectional study with stratified and cluster sampling, evaluating anxiety symptoms (AS) and depression symptoms (DS) in 1637 adults. Vitamin D levels were measured using electrochemiluminescence and were considered deficient when < 20 ng/mL in a healthy population or < 30 ng/mL in at-risk groups. Genotyping was performed using real-time polymerase chain reaction with TaqMan probes. The prevalence rates of AS, DS, and vitamin D deficiency were 23.5%, 15.8%, and 30.9%, respectively. No direct association was observed between vitamin D deficiency and AS or DS. However, interaction analysis revealed a combined effect of vitamin D deficiency and FokI for DS but not for AS. Individuals with vitamin deficiency and one or two copies of the altered allele of the FokI exhibited a higher prevalence of DS than individuals homozygous for the wild-type allele and vitamin D sufficiency. The interaction between vitamin D deficiency and the FokI polymorphism was associated with DS.

## Introduction

Vitamin D belongs to the steroid hormone family and plays a role in calcium metabolism and bone homeostasis^1^. Another less-explored function of vitamin D is related to extraskeletal effects, such as cell growth, neuromuscular and immune function^2^, brain development, neuroprotection, regulation of neurotrophic factors, neurotransmission, and synaptic plasticity^3^, which may have an impact on developing mental disorders.

Vitamin D exists in two primary forms: ergocalciferol (vitamin D2) and cholecalciferol (vitamin D3). Vitamin D2 is obtained from limited dietary sources, including mushrooms, fortified foods, and supplements^4^. In contrast, vitamin D3 is primarily produced in the human body through a photochemical reaction in the skin upon exposure to ultraviolet B radiation^4,5^. However, vitamin D3 can also be obtained in small quantities from animal-based foods, including milk, eggs^5^, fish, and cod liver oil^4^.

Vitamin D metabolism begins in the liver, where the first hydroxylation process occurs to form 25-hydroxyvitamin D (25(OH)D) (calcidiol)^4,6^, which is a biomarker of serum vitamin D levels^4^. Subsequently, vitamin D binding protein transports 25(OH)D from the liver to the kidneys, where the primary hydroxylation occurs, converting 25(OH)D into 1-α-25-dihydroxy vitamin D (calcitriol)^6^, which is the biologically active form of the vitamin. This active form is responsible for most biological functions of vitamin D^7^.

One of the mechanisms explaining the association between serum vitamin D levels and mental disorders is the involvement of vitamin D in serotonin metabolism and its effects on circadian rhythms^8^. In the body, the function of vitamin D is mediated by its binding to the vitamin D receptor (VDR), a member of the nuclear hormone receptor family. The binding of active vitamin D to the VDR leads to the formation of a transcriptional complex^9^ modulating the gene transcription related to vitamin D function, such as CYP24A1, PTH, and FGF23^10^. VDR is expressed in different cells^4^ and tissues, including neurons, glial cells, and specific brain regions, and has been implicated in the pathophysiology of mental disorders^8,11^.

Genetic variants, such as the single nucleotide polymorphism (SNP) FokI (rs2228570), can affect the responsiveness of VDR^12^. FokI is located in exon 2 of the VDR gene and modifies its transcription initiation site^7^. This is the only variant associated with a change in the length of the encoded protein^2^, resulting in a less active protein^7,13^. This polymorphism alters the efficiency of vitamin D binding^9^ and reduces the receptor’s transcriptional capacity^7,13^.

Therefore, it may be postulated that this alteration in the VDR protein can result in functional consequences similar to those of vitamin D deficiency. Although the FokI polymorphism has been investigated in relation to major depressive disorder^11^, cognitive functioning, depressive symptoms^14^, and self-reported depression^12^, it has not been studied in relation to anxiety. Moreover, these studies were conducted in other countries and do not necessarily apply to the Brazilian population, which is characterized by miscegenation^15^ and high sun exposure^16^. Therefore, there is a pressing need for studies that investigate this association in other contexts.

Vitamin D deficiency can impact anxiety symptoms (AS) and depression symptoms (DS). Additionally, the high prevalence of this deficiency, even in tropical countries such as Brazil^16,17^, could have been exacerbated by social isolation during the coronavirus 2019 (COVID-19) pandemic. Therefore, it is crucial to investigate the factors involved in vitamin D metabolism and their association with mental disorders.

We hypothesized that vitamin D deficiency is associated with AS and DS, and the presence of the VDR gene polymorphism (rs2228570) FokI has an interactive effect on this association. Thus, this study aimed to investigate the association between vitamin D deficiency and AS and DS in adults while assessing the role of the FokI polymorphism.

## Materials and methods

### Study design

This investigation is part of the “Epidemiological surveillance of COVID-19 in the Inconfidentes Region/MG” (COVID-Inconfidentes Study). The aim was to assess the prevalence of SARS-CoV-2 infection during the first wave of the pandemic and its subsequent socioeconomic and health impacts in two medium-sized cities, Ouro Preto and Mariana, located in the state of Minas Gerais, Brazil. The COVID-Inconfidentes Study was a population-based cross-sectional survey conducted between October and December 2020, corresponding to the spring season in Brazil. The sample size was calculated using the OpenEpi tool (https://www.openepi.com/Menu/OE_Menu.htm) based on the 2010 population census^18^ for the urban area of each city, adopting a 95% confidence level (95% CI), design effect equal to 1.5, and 20% recomposition percentage for any loss. After performing sample calculations, we determined that a minimum of 1464 individuals were required for the study.

A three-stage conglomerate sampling design was adopted (census, household, and residential). For each city, strata were defined according to the average monthly income in the 2010 census to ensure the representativeness of the different socioeconomic strata in the sample^18^. The sample weight for each selection unit was calculated according to the methodology described by Silva et al.^19^.

### Data collection

Data were collected through face-to-face interviews conducted by trained interviewers who applied a structured electronic questionnaire using the Data Goal application. Based on previously validated instruments, the questionnaire contained sociodemographic characteristics, lifestyle, health conditions, and diet questions. Blood samples were collected for biochemical and genetic analyses. Because of in-person data collection, all recommendations set by the national protocols to contain the spread of COVID-19 were adopted, including the use of personal protective equipment.

Detailed information about the methodology and questionnaire can be accessed in the study by Meireles et al.^20^.

### Analytic sample

A total of 1789 participants, including individuals of both sexes, were selected for the survey. Individuals who did not answer the questionnaire (n = 9), those with incomplete information in the scales assessing the study outcomes (n = 69), those whose answers contained no information on sample weight calculations (n = 18), and those with insufficient blood samples for biochemical and genetic analyses were excluded from the study (n = 56). Therefore, the final sample consisted of 1,637 participants.

### Measures

#### Outcome: symptoms of mental disorders

AS and DS were measured using the 7-item Generalized Anxiety Disorder (GAD-7) and 9-item Patient Health Questionnaire (PHQ-9) scales, both of which have been validated in Brazil^21–23^. The scales assess negative symptoms related to mental disorders in the last 2 weeks. The GAD-7 score ranges from 0 to 21 points, and the PHQ-9 score ranges from 0 to 27. This study defined the presence of AS and DS when the score obtained was ≥ 10^24,25^.

#### Biological sample

Blood was collected by puncturing the cubital fossa region and performed by a trained professional according to protocol. Two samples were obtained and stored in two tubes: a 7.5 mL S-Monovette^®^ (Sarstedt AG & Co., Nümbrecht, Germany) serum gel for vitamin D analysis and a 2.7 mL S-Monovette^®^ (Sarstedt) K3 EDTA for genetic analysis.

The samples were checked, stored in a cool box, and transported to the Epidemiology Laboratory of the Medical School of the Federal University of Ouro Preto. Subsequently, the samples with serum gel were centrifuged at 2500 rpm for 15 min, and aliquots were prepared and stored in a freezer at − 80 °C for the biochemical analysis. Samples containing EDTA were stored at − 20 °C until genetic analyses were performed.

#### Exposure variable: serum vitamin D levels

The serum 25(OH)D level was determined by indirect electrochemiluminescence in the Access 2 Immunoassay System (Beckman Coulter, USA) using the commercial Roche Diagnostics kit (Roche, Switzerland). During the intralaboratory analysis, the method displayed a coefficient of variation ranging from 6.1 to 7.5%, with the observed value in our sample being 1.77%. Furthermore, the correlation coefficient with liquid chromatography with tandem mass spectrometry considered the gold standard, was 0.92, as provided by the manufacturer. For our analyses, the serum levels were evaluated as continuous and categorical variables. They were classified according to the Brazilian Society of Metabolism and Endocrinology as deficient when the levels of 25(OH)D were < 20 ng/mL in the healthy population or < 30 ng/mL for the at-risk groups, such as older adults, pregnant and lactating women, patients with rickets/osteomalacia, osteoporosis, patients with a history of falls and fractures, secondary causes of osteoporosis, hyperparathyroidism, inflammatory diseases, autoimmune diseases, chronic kidney disease, and malabsorption syndromes^26^.

#### Interaction variable: polymorphism FokI

To detect the FokI VDR gene polymorphism (rs2228570), genomic DNA was extracted from venous blood samples. Per the manufacturer’s protocol, a 600 µL whole blood sample was thawed and used immediately with the Wizard Genomic DNA Purification kit (Promega, USA). After extraction, the DNA was stored in a hydration solution at 4 °C for 24 h. Then, the samples were stored at − 20 °C until quantification, which was performed using fluorometry (Fluorometer Qubit 2.0, Invitrogen) and genotyping.

Polymorphism detection was performed using DNA fragment amplification by the TaqMan SNP Genotyping Assay (Applied Biosystems, Foster City, CA, USA) of real-time polymerase chain reaction (PCR) (initial denaturation: 95 °C, 10 min; ringing: 40 cycles of 95 °C, 15 s; final extension: 60 °C, 1 min) using specific primers from Thermo Scientific (Assay ID: 12060045_20).

Genotyping was performed using TaqMan allelic discrimination with sequence detection software on the 7500 Fast Real-Time PCR System (Applied Biosystems, Foster City, CA, USA), according to the manufacturer’s instructions, to detect the transition substitution A > G. Negative controls were used in all experimental runs to ensure quality control and identify any potential contamination. When conflicting interpretations arose, the samples underwent supplementary amplification for confirmation, as required. Importantly, it is worth noting that the SNP conformed to the Hardy–Weinberg equilibrium (*P* > 0.05). Individuals were classified as homozygous wild-type (AA), heterozygous (AG), or homozygous mutant (GG).

#### Descriptive variables and covariates

The following variables were assessed: age; sex (female, male); skin color (white or non-white); education level (no schooling, ≤ 9 years of schooling, > 9 years of schooling); work status (unemployed/housework, currently employed); work routine during the pandemic (worked outside, no work, worked from home); social restriction (yes, no); current alcohol consumption (yes, no); smoking (yes, no); vitamin D supplementation (yes, no); self-reported chronic physical pain, considering physical pain present for ≥ 3 months (yes, no); and self-rated health (fair/poor/very poor/very good/good). Reported morbidity was assessed through the question, “Has any doctor or other health professional ever informed you of having a chronic disease?” Individuals who responded affirmatively were categorized as having a reported morbidity. The available response options included chronic diseases such as hypertension, diabetes, asthma, bronchitis, chronic lung disease, cancer (any type), chronic kidney disease, heart disease, depression, anxiety disorder, sleep apnea, hypo- or hyperthyroidism, others, and none of the above. In addition, the COVID-19 diagnosis was evaluated using the Wondfo SARS-CoV-2 Antibody Rapid Test (seropositive, seronegative). Sleep quality (good, poor) was assessed using the Pittsburgh Sleep Quality Index questionnaire, with scores ≥ 5 classified as poor sleep quality^27^. Current family income was categorized according to the number of minimum wages received (≤ 2 minimum wages or up to 388.5 US Dollars (USD); > 2 and ≤ 4 minimum wages or greater than 388.5 USD, up to 770.0 dollars; > 4 minimum wages or greater than 770.0 USD). In Brazil, the minimum wage is the lowest salary employers can legally pay their employees and is used as a national reference.

Physical activity (dichotomous nominal variable, inactive or active) was assessed. Those who had at least 150 min per week of moderately intense physical activity or at least 75 min per week of vigorously intense physical activity were classified as active^28^.

Sun exposure (dichotomous nominal variable, sufficient or insufficient) was categorized according to the average daily sun exposure: [total time of daily sun exposure (min) × frequency of sun exposure weekly/7]. This was then classified as “insufficient sun exposure” if < 30 min per day^29^. Finally, body mass index (BMI) (dichotomous nominal variable, not overweight or overweight) was classified as not overweight (BMI < 25 kg/m^2^ for adults and < 27 kg/m^2^ for older adults) and overweight (BMI ≥ 25 kg/m^2^ for adults and ≥ 27 kg/m^2^ for older adults), according to the World Health Organization (WHO)^30^ for adults and Lipschitz^31^ for older adults, respectively.

### Statistical analysis

Categorical variables were described as relative frequencies and 95% CI, and continuous variables were described as means and 95% CI.

**Figure 1:**
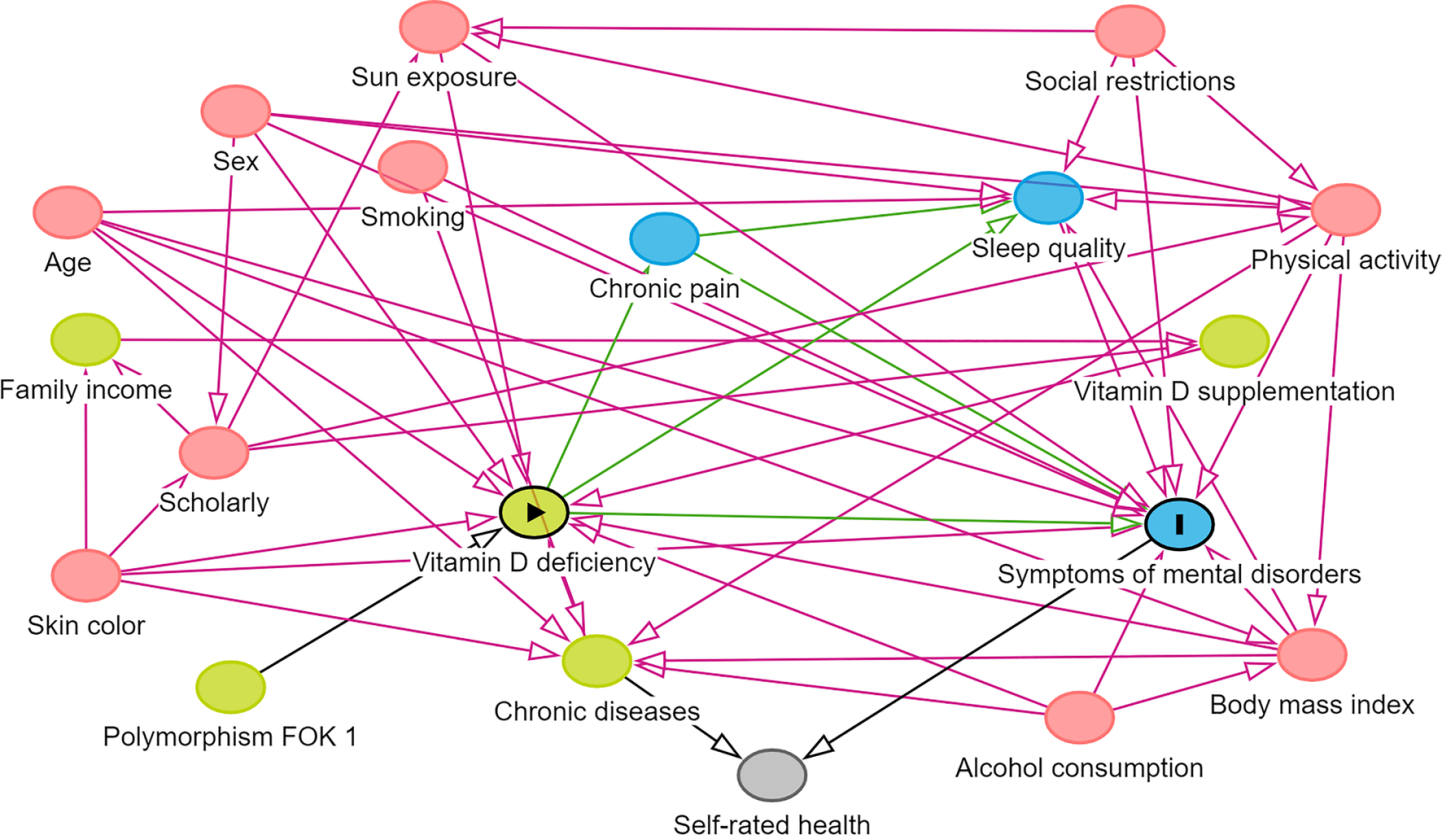
Directed acyclic graph (DAG) on the influence of the FokI polymorphism on the association between vitamin D deficiency and symptoms of anxiety and depression in adults. Legend: Causal connections represented by arrows were established between variables. The variable in green and with the “►” symbol was the exposure variable; in blue and with the letter “I” was the response variables; variables in blue are the antecedents of the outcome variable; and those in red are antecedents of the outcome and exposure variables; the variable in gray is a collider and indicates a closed or blocked path.

To guide the analysis, a causality model based on a directed acyclic graph (DAG) was developed with the exposure variable (vitamin D deficiency), outcomes (AS and DS), the interaction variable (polymorphism FokI), and possible confounding variables (Fig. 1) using the online software Dagitty version 3.2^32^. A t-test was performed to verify differences in means. Crude and adjusted Poisson regressions were performed for the variables indicated by the DAG. Therefore, the model was fitted using the minimum variables: sex, age, skin color, alcohol consumption, BMI, physical activity, sun exposure, chronic disease, and social restrictions.

In addition, multiplicative interaction analysis was performed to verify how FokI interfered with the association between exposure and outcomes. The same adjustment was adopted for these analyses, except for the association between FokI and the outcomes, which was adjusted only for sex and age.

A power calculation using OpenEpi demonstrated that this study achieved 100% statistical power at a 95% confidence level. All statistical analyses were performed considering a complex sample design using the “svy” package of Stata software, version 13.1 (version 13.1; Stata Corporation, College Station, TX, USA) with a statistical significance of 5%.

### Ethical considerations

All procedures followed the Brazilian standards for research involving human beings, the Declaration of Helsinki, and the seroepidemiological investigation protocol for SARS-CoV-2 infection by the WHO^33^. This study was approved by the Research Ethics Committee of the Federal University of Ouro Preto (Ethics Submission Certificate No. 32815620.0.1001.5149). Written informed consent was obtained from all the participants.

## Results

### Study population

**Figure 2:**
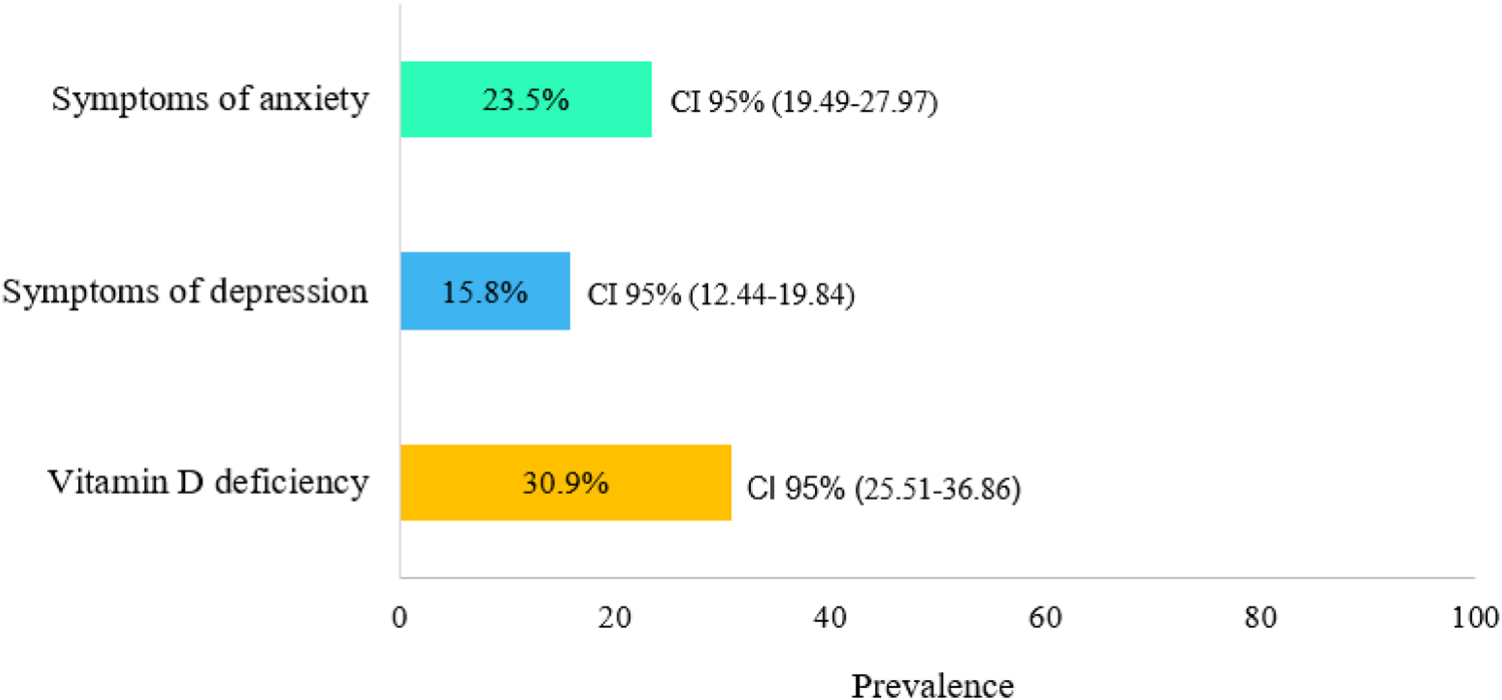
Prevalence rates for anxiety symptoms, depression symptoms, and vitamin D deficiency in the sample, COVID-Inconfidentes Study (2020).

**Figure 3:**
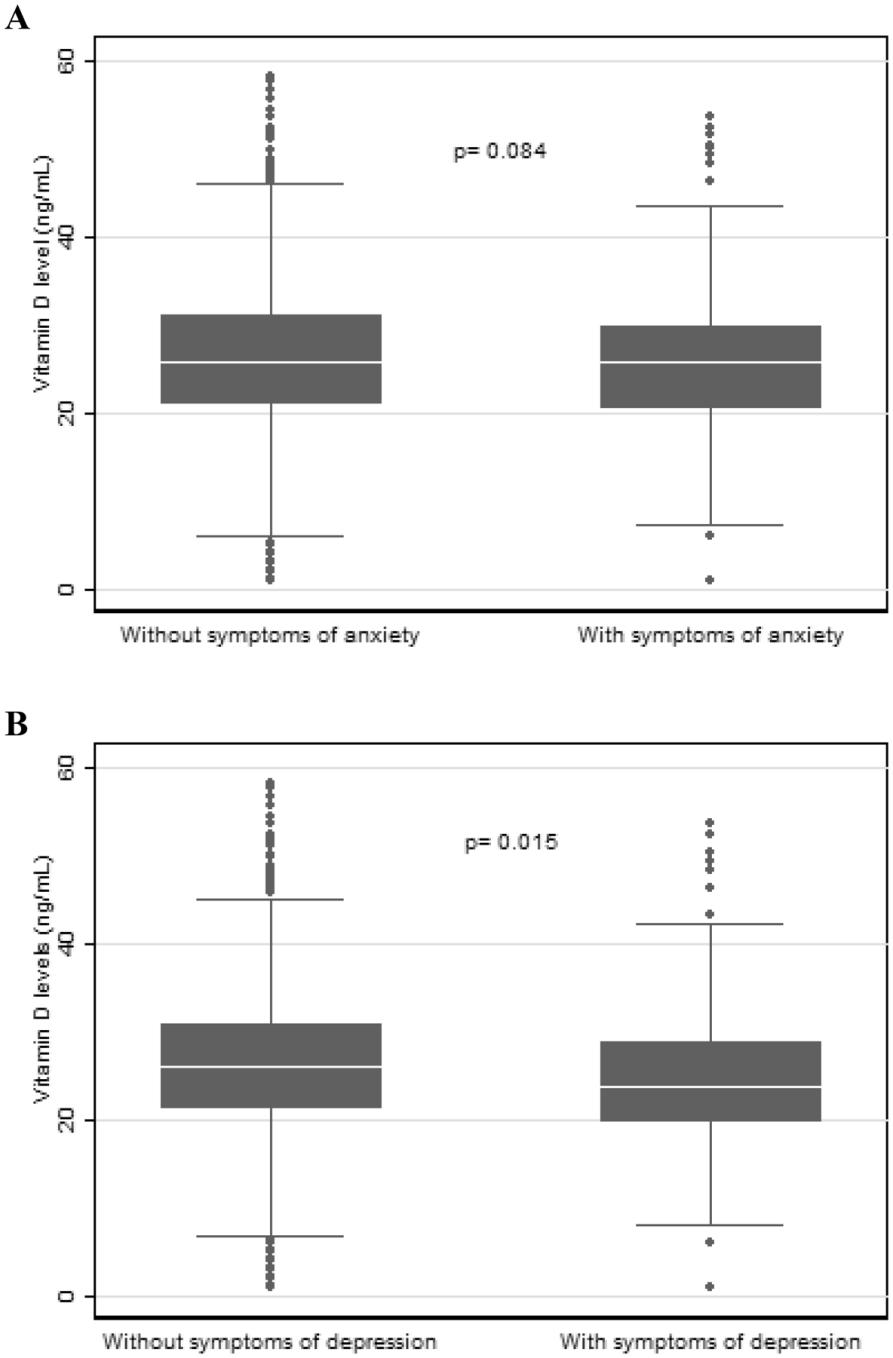
Vitamin D levels (ng/mL) according to the symptoms of anxiety (**A**) and symptoms of depression (**B**).

Among the 1,637 participants, 50.9% were women with a mean age of 42.9 years. Most participants had > 9 years of schooling, were employed, and had a family income of ≤ 2 minimum wages or up to 388.5 USD. Regarding skin color, most identified as non-white (Table 1). Among the participants, 23.5% reported having AS, 15.8% reported DS, and 30.9% had vitamin D deficiency (Fig. 2). No difference was observed in the mean vitamin D levels among individuals with AS (without AS: mean, 26.5; standard error (SE): 0.55; 95% CI 25.40–27.59 vs. with AS: mean, 25.3; SE: 0.61; 95% CI 24.05–26.48; *P* = 0.084). However, these levels were slightly lower in participants with depression (without DS: mean, 26.5; SE: 0.52; 95% CI 25.51–27.58 vs. with DS: mean, 24.4; SE: 0.69; 95% CI 23.06–25.81; *P* = 0.015) (Fig. 3).

**Table 1:** Socioeconomic, lifestyle and health conditions in adults according to vitamin D status during the COVID-19 pandemic, COVID-Inconfidentes Study (2020).

Characteristics	Total	Vitamin D status		PR	95% CI	*p* Value *
		Deficiency (%)	Sufficiency (%)			
		30.9	69.1			
Age (years)	Mean (CI 95%)	Mean (CI 95%)	Mean (CI 95%)			
	42.9 (41.62–44.26)	51.0 (48.12–53.95)	39.8 (38.36–41.14)	1.03	1.02; 1.04	** < 0.001**
Sex	% (CI 95%)	% (CI 95%)	% (CI 95%)			
Female	51.3 (43.76–58.71)	54.9 (47.48–62.06)	49.6 (39.78–59.54)	1 (Ref.)		
Male	48.7 (41.29–56.24)	45.1 (37.94–52.52)	50.4 (40.46–60.22)	0.86	0.62; 1.20	0.386
*Family income”*						
≤ 2 MW^a^	38.6 (33.37–44.15)	35.7 (26.11–46.60)	40.0(34.03–46.19)	1 (Ref.)		
> 2 to ≤ 4 MW	33.4 (27.86–39.50)	38.8 (29.97–48.39)	31.0 (24.09–38.84)	1.25	0.82; 1.93	0.296
> 4 MW	28.0 (22.55–34.07)	25.5 (18.87–33.52)	29.0 (22.77–36.28)	0.99	0.68; 1.43	0.946
*Skin color*						
White	26.4 (20.97–32.71)	30.4 (21.66–40.90)	24.6 (18.60–31.84)	1 (Ref.)		
Black/brown	73.6 (67.29–79.03)	69.6 (59.10–78.34)	75.4 (68.16–81.40)	0.82	0.57; 1.18	0.285
*Education level (years of schooling)*						
No schooling	1.6 (0.68–3.81)	4.4 (1.68–10.99)	3.8 (0.16–0.94)	1 (Ref.)		
≤ 9	26.4 (21.63–31.81)	34.0 (27.47–41.12)	23.0 (16.71–30.85)	0.47	0.34; 0.66	** < 0.001**
> 9	72.0 (66.57–76.80)	61.6 (54.04–68.70)	76.6 (68.8–82.9)	0.32	0.24; 0.42	** < 0.001**
*Work status*						
Unemployed/housework	45.8 (41.28–50.34)	54.4(46.53–62.05)	41.9 (36.38–47.69)	1 (Ref.)		
Currently employed	54.2 (49.66–58.72)	45.6 (37.95–53.47)	58.1 (52.31–63.62)	0.71	0.54; 0.92	**0.011**
*Work routine during the pandemic*						
No work from home	32.8 (27.81–38.27)	28.3 (19.80–38.61)	34.9 (29.77–40.33)	1 (Ref.)		
No work	21.4 (17.12–26.41)	17.3 (12.45–23.68)	23.2 (23.21–29.47)	0.94	0.61; 1.44	0.782
Work from home	45.8 (41.28–50.34)	54.4 (46.51–62.02)	41.9 (36.38–47.69)	1.38	0.97; 1.95	0.069
*Social restriction*						
No	14.02 (10.19–18.97)	15.97 (8.32–28.47)	13.1 (9.69–17–57)	1 (Ref.)		
Yes	85.98 (81.03–89.81	84.03 (71.53–91.68)	86.9 (82.43–90.31)	0.86	0.51; 1.43	0.551
*Alcohol consumption*						
No	41.0 (34.80–47.47)	51.8 (42.99–60.49)	36.1 (29.10–43.82)	1 (Ref.)		
Yes	59.0 (52.53–65.20)	48.2 (39.51–57.01)	63.9 (56.18–70.90)	0.65	0.47; 0.89	**0.008**
*Smoking*						
No	83.9 (78.89–87.83)	80.5 (69.17–88.35)	85.4 (80.02–89.47)	1 (Ref.)		
Yes	16.1 (12.17–21.11)	19.5 (11.65–30.83)	14.6 (10.53–19.98)	1.26	0.79; 2.00	0.325
*Physical activity*^*b*^						
Active	36.1 (28.48–44.52)	31.1 (22.25–41.68)	38.3 (28.24–49.50)	1 (Ref.)		
Inactive	63.9 (55.48–71.52)	68.9 (58.32–77.75)	61.7 (50.50–71.76)	1.25	0.77; 2.02	0.357
*Sun exposure*^*c*^						
Sufficient	41.4 (34.04–49.09)	38.2 (28.38–49.09)	42.8 (33.17–52.96)	1 (Ref.)		
Insufficient	58.8 (50.91–65.96)	61.8 (50.91–71.62)	57.2 (47.04–66.83)	1.14	0.73; 1.77	0.554
*Vitamin D supplementation*						
No	93.3 (91.23–94.89)	95.1 (92.68–96.85)	92.4 (89.49–94.61)	1 (Ref.)		
Yes	6.7 (5.11–8.77)	4.9 (3.15–7.32)	7.6 (5.39–10.51)	0.70	0.45; 1.09	0.115
*Chronic disease*^*d*^						
No	42.3 (35.22–49.75)	21.4 (15.59–28.58)	51.7 (42.75–60.50)	1 (Ref.)		
Yes	57.7 (50.25–64.78)	78.6 (71.42–84.41)	48.3 (39.50–57.25)	2.70	1.76; 4.12	** < 0.001**
*Chronic pain*^*e*^						
No	65.8 (61.23–70.13)	63.1 (56.90–68.97)	67.0 (60.66–72.80)	1 (Ref.)		
Yes	34.2 (29.87–38.77)	36.9 (31.03–43.10)	33.0 (27.20–39.34)	1.12	0.87; 1.46	0.373
*Self-rated health*^*f*^						
Very good/good	79.3 (75.12–83.00)	66.7 (60.26–72.6)	85.0 (80.04–88.88)	1 (Ref.)		
Fair/poor/very poor	20.7 (17.00–24.88)	33.3 (27.40–39.74)	15.01 (11.12–19.96)	1.92	1.47; 2.49	** < 0.001**
*Sleep quality*^*g*^						
Good	46.2 (41.93–50.57)	42.1 (33.38–51.32)	48.1 (43.35–52.83)	1 (Ref.)		
Poor	53.8 (49.43–58.07)	57.9 (48.68–66.62)	51.9 (47.17–56.65)	1.18	0.88; 1.58	0.257
*COVID-19 Test*						
Soronegative	95.0 (93.22–96.26)	94.7 (91.22–96.86)	95.1 (92.56–96.75)	1 (Ref.)		
Soropositive	5.0 (3.74–6.78)	5.3 (3.14–8.78)	4.9 (3.25–7.44)	1.05	0.62; 1.76	0.851
*Genotype frequency (FokI)*^*h*^						
AA	9.9 (5.53–16.77)	6.7 (4.47–9.82)	11.2 (5.54–21.34)	1 (Ref.)		
AG	44.7 (40.59–48.96)	45.7 (37.11–54.62)	44.3 (40.09–48.58)	1.50	0.76; 2.97	0.237
GG	45.4 (39.41–51.65)	47.6 (39.47–55.87)	44.5 (36.70–52.59)	1.54	0.73; 3.25	0.253

*PR* prevalence ratio (crude). ^a^ MW: minimum wage. "Family income: minimum wage value (2020): BRL 1045.00 ≈ USD 194.25 (1 USD = 5.3797 BRL). ^b^ Physical activity—active: ≥ 150 min of moderate physical activity or ≥ 75 min of vigorous physical activity (WHO—World Health Organization, 2010). ^c^ Sun exposure: insufficient sun exposure < 30 min/day. ^d^ Chronic disease: medical diagnosis of at least one chronic disease. ^e^ chronic physical pain, considering physical pain present for 3 months or more (yes, no). ^f^ Self-rated health: fair/poor/very poor health or very good, good. ^g^ Sleep quality: scores of 0–4 = good sleep quality; scores ≥ 5 = poor sleep quality. ^h^ Genotype frequency (FokI): AA—homozygous wild, AG—heterozygous and GG—homozygous mutant. *p value from univariate Poisson regression. Bold values indicate statistical significance of 5%.

### Vitamin D deficiency-associated factors

Vitamin D deficiency was positively associated with older age, chronic disease diagnosis, and worse self-rated health. In contrast, vitamin D deficiency was negatively associated with educational level, work status, and alcohol consumption (Table 1).

### Vitamin D deficiency, mental disorders, and FokI polymorphism

In the multivariate model, no association was observed between vitamin D deficiency and AS or DS. Similarly, no association was observed between the polymorphism and outcomes. However, in the interaction analyses, a combined effect between vitamin D deficiency and the presence of the altered allele was observed for DS but not for AS (Table 2). Individuals with vitamin D deficiency and the presence of one altered allele had a 2.84 higher prevalence of DS (95% CI 1.04–7.76), while individuals with the presence of two altered alleles had a 4.37 higher prevalence of DS (95% CI 1.62–11.83) (Table 2).

**Table 2:** Association between vitamin D deficiency and symptoms of anxiety and depression during the COVID-19 pandemic, and influence of polymorphism FokI (rs10735810) in the VDR gene in this association; COVID-Inconfidentes Study (2020).

	Symptoms of anxiety		*p* Value*	Symptoms of depression		*p* Value*
	PR	(95% CI)		PR	(95% CI)	
*Vitamin D*						
Suficiency	1 (Ref)			1 (Ref)		
Deficiency (Crude analysis)	0.97	(0.71–1.32)	0.842	1.47	(0.93–2.33)	0.101
Deficiency (Adjusted analysis)	1.02	(0.77–1.35)	0.882	1.31	(0.85–2.02)	0.225
*Polymorphism FokI*						
AA	1 (Ref)			1 (Ref)		
AG	0.98	(0.31–3.15)	0.980	0.76	(0.28–2.08)	0.588
GG	0.97	(0.38–2.48)	0.949	0.64	(0.24–1.74)	0.381
*Adjustment*^*b*^						
AA	1 (Ref)			1 (Ref)		
AG	0.90	(0.33–2.43)	0.832	0.64	(0.30–1.35)	0.239
GG	0.89	(0.41–1.93)	0.770	0.54	(0.27–1.08)	0.080
*Crude interaction analysis*						
Vitamin D sufficiency * AA	1 (Ref)			1 (Ref)		
Vitamin D deficiency * AG	0.65	(0.12–3.49)	0.613	4.57	(1.01–20.73)	**0.049**
Vitamin D deficiency * GG	2.06	(0.52–8.23)	0.301	5.39	(1.24–23.34)	**0.025**
*Adjusted interaction analysisˠ*						
Vitamin D sufficiency * AA	1 (Ref)			1 (Ref)		
Vitamin D deficiency * AG	0.65	(0.17–2.53)	0.531	2.84	(1.04–7.76)	**0.042**
Vitamin D deficiency * GG	2.32	(0.74–7.33)	0.149	4.37	(1.62–11.83)	**0.004**

*PR* prevalence ratio. The exposure variable was vitamin D deficiency (25(OH)D < 20 ng/mL in a healthy population or < 30 ng/mL for groups at risk). The outcome variables were symptoms of anxiety and symptoms of depression, evaluated by GAD-7 and PHQ-9, respectively. Polymorphisms were genotyped using Taqman probes in real time PCR, and were classified as homozygous wild (AA), heterozygous (AG), and homozygous mutant (GG). Directed acyclic graph (DAG) was used for the adjusted analysis in the association between vitamin D and symptoms of anxiety and depression. Adjusted for minimum and sufficient set of variables: sex, age, skin color, body mass index, physical activity, alcohol consumption, sun exposure, chronic disease, and social restriction. *ˠ*Adjusted Interaction analysis: adjusted for minimum and sufficient set of variables. ^b^Adjustment (exclusive for association between polymorphism and symptoms of anxiety and depression): sex and age. *p value from Poisson regression. Bold values indicate statistical significance of 5%.

## Discussion

This study was conducted in Brazil to explore whether the VDR gene polymorphism FokI (rs2228570) influences the association between vitamin D deficiency, AS, and DS in adults. Our main findings were that vitamin D deficiency and the presence of the FokI polymorphism alone were not associated with AS or DS. However, a combined effect was observed between the AG and GG genotypes and vitamin D deficiency in relation to DS but not to AS. Notably, the prevalence of DS was higher among individuals with two copies of the altered allele.

Vitamin D deficiency is highly prevalent globally and has been found in different populations, such as children, adolescents, adults, and older adults, irrespective of race, ethnicity, or country^34^. Low serum vitamin D levels are associated with depressive symptoms^8,35,36^, which was not observed in our study. A meta-analysis of observational studies indicated that individuals with lower vitamin D levels were 1.31 times more likely to experience depression (95% CI 1.0–1.71, *P* = 0.05) and had a 2.21 higher risk of depression (95% CI 1.40–3.49, *P* = 0.0007)^36^. However, these findings and those of other studies should be approached with caution. Consideration of potential biases in study design, non-representative samples, and variations in the definition of vitamin D deficiency^36^ is crucial. Methodological disparities might have contributed to variations in study outcomes. Moreover, limitations in the existing literature, including diverse study populations, variations in depression severity, distinct methods for measuring 25(OH)D^37^, and diverse measures of depression^38^, could explain the disparities between the published results and the outcomes of our study. Although we did not find an association between serum vitamin D levels and AS or DS, we observed that individuals have lower mean serum vitamin D levels. A bidirectional relationship is possible between lower serum vitamin D levels and mental health disorders. That is, lower vitamin D levels can lead to the development of mental health disorders. However, individuals with mental health disorders may be socially isolated and have reduced exposure to sunlight, so the opposite can be true^39^.

Regarding anxiety, the association between low serum vitamin D levels and AS has been explored in a small number of studies and specific subgroups^40–42^. However, the results of a cross-sectional study demonstrated a negative association between serum vitamin D levels and a self-reported state of anxiety (OR = 0.92, *P* < 0.001)^1^.

Various mechanisms have been proposed to explain the association between serum levels and mental health disorders. One of these involves the role of vitamin D in serotonin metabolism and its interaction with circadian rhythms^8^. An experimental study showed that sufficient serum vitamin D levels enhanced the production of serotonin and suppressed its reuptake and catabolism^43^. Serotonin is a neurotransmitter that plays a central role in the pathogenesis of anxiety and depression^44^, making it a plausible mechanism for the link between vitamin D and mental health disorders. Furthermore, serotonin is a precursor of melatonin, a hormone involved in regulating circadian rhythms and associated with sleep quality^8^. Thus, low vitamin D levels can compromise sleep quality and play a fundamental role in the symptomatology of anxiety and depression^8,45^.

In addition to the aforementioned pathways, other plausible mechanisms link vitamin D to mental health disorders. Vitamin D regulates the detoxification process in the brain by controlling γ-glutamyl transpeptidase activity. Vitamin D also regulates the expression of neuronal growth factors such as neurotrophin-3 and neurotrophin-4^46^ and the secretion of brain-derived neurotrophic factor^47,48^. Additionally, vitamin D acts by reducing neuronal calcium levels. Consequently, lower levels of vitamin D may result in an imbalance between excitatory and inhibitory neurons that can contribute to developing mental health disorders^49^.

As previously mentioned, the function of vitamin D in target tissues is mediated by its binding to the VDR^9^. The disparities in the affinity of vitamin D for VDR can be attributed to the presence of polymorphisms in the VDR gene, especially FokI^12^. Our study did not demonstrate a direct association between the genotypes of the FokI polymorphism and AS or DS. Similarly, a cross-sectional study conducted with 86 patients diagnosed with major depressive disorder in Turkey showed no association between depression and the FokI polymorphism^11^. The results of a prospective population-based study conducted with 563 participants in The Netherlands showed that polymorphisms in the VDR gene, such as BsmI (rs1544410) and TaqI (rs731236), but not FokI, influence the susceptibility to age-related changes in cognitive functioning and depressive symptoms^14^. A study conducted on 153 Ukrainian patients with different types of thyroid pathology did not reveal an association between the presence of this genetic alteration and anxiety^50^. Therefore, it is important to evaluate the presence of polymorphisms in combination with other factors, such as serum vitamin D levels.

A noteworthy result of our study was the interaction observed between vitamin D deficiency and the FokI polymorphism in DS. While isolated factors were not associated with symptoms, their combination was linked to a higher prevalence of DS. These findings highlight the complex and multifactorial nature of mental health disorders, which involve a combination of social, economic, environmental, and biological factors^51–53^. Notably, the observed association was exclusively found in carriers of the altered allele (G), suggesting that individuals with wild-type homozygotes, whose receptor activity is not impaired and are known to be more efficient in mediating the action of vitamin D^54^, did not show an association between low vitamin D levels and disorder symptoms. However, in the presence of at least one altered allele (and, as a result, lower binding of vitamin D to its receptor), the deficiency was linked to a higher prevalence of DS. Therefore, these findings imply that individuals with an altered allele should be considered to have higher vitamin D levels owing to their potential influence on DS.

Considering the high prevalence of mental health disorders and vitamin D deficiency, their potential association is a major public health concern. Public and mental healthcare policies should incorporate nutritional strategies as part of preventive or treatment interventions, particularly for depression. Therefore, maintaining adequate serum vitamin D levels is crucial. Sun exposure can achieve this; however, the current literature lacks specific recommendations. Alternatively, it can also be achieved through vitamin D supplements, which is an economical strategy without significant adverse effects^55^.

This study has some limitations. First, the cross-sectional design did not allow us to assess the direction of causality. Residual confounders, owing to unmeasured factors, cannot be completely excluded. Additionally, gene–gene and gene-environment interactions should be the focus of further scientific investigation when studying associations such as those proposed in our study. Although our current study provides valuable insights, more advanced techniques, such as RNA sequencing and a panel of SNPs, can offer a deeper understanding of these interactions. Considering broader analyses is crucial for future research to seek a more nuanced perspective on the relationships explored. Finally, it is important to note that the results and prevalence observed in this study should not be generalized to other populations.

To our knowledge, this is the first population-based study to evaluate the association of vitamin D deficiency and FokI polymorphism on mental disorder symptoms in adults. In addition, probabilistic sample selection and sample weight calculations provided statistical power to the study as well as internal and external validity. Genetic marker evaluation in a large sample size and using DAG to avoid unnecessary adjustments were also essential aspects of this study.

## Conclusion

Although vitamin D deficiency and the FokI polymorphism were not individually associated with AS and DS, a combined effect exists stemming from the interaction of these two factors in relation to DS. Individuals with vitamin D deficiency and at least one altered allele were more likely to have higher DS prevalence.

## Data Availability

The datasets generated during and/or analysed during the current study are not publicly available due to confidentiality agreements with participants but are available from the corresponding author on reasonable request.
